# Diagnostic performance of the Luminex xTAG gastrointestinal pathogens panel to detect rotavirus in Ghanaian children with and without diarrhoea

**DOI:** 10.1186/s12985-016-0588-1

**Published:** 2016-07-29

**Authors:** Amelie Leva, Daniel Eibach, Ralf Krumkamp, Julia Käsmaier, Dennis Rubbenstroth, Yaw Adu-Sarkodie, Jürgen May, Egbert Tannich, Marcus Panning

**Affiliations:** 1 Institute for Virology, Center for Microbiology and Hygiene, University Medical Center – Freiburg University, Hermann-Herder-Str. 11, 79104 Freiburg, Germany; 2 Bernhard Nocht Institute for Tropical Medicine, Bernhard-Nocht-Str. 74, 20359 Hamburg, Germany; 3 German Center for Infection Research (DZIF), Hamburg-Borstel-Lübeck, Hamburg, Germany; 4 Kwame Nkrumah University of Science and Technology (KNUST), Kumasi, Ghana

**Keywords:** Rotavirus, Luminex xTAG gastrotinestinal pathogens panel, Stool samples, Performance, Sub-saharan Africa

## Abstract

**Background:**

Rotavirus is one of the leading causes of childhood diarrhoea worldwide. The highest disease burden is seen in resource-constrained settings of sub-Saharan Africa. Recently, commercial multiplex PCR panels proved their accuracy to diagnose infectious gastroenteritis in Europe and the USA. However, data on their performance using samples from tropical regions in general and to detect rotavirus in particular remains scant. We aimed to analyse the diagnostic performance of the Luminex xTAG gastrointestinal pathogens panel, a multiplex PCR, to detect rotavirus in stool samples from Ghanaian children.

**Methods:**

A total of 682 stool samples were collected in the Ashanti region of Ghana between 2007 and 2008. Of these, 341 were from cases (children with diarrhoea), and another 341 from controls (children without diarrhoea). All samples were analysed using the Luminex xTAG assay and compared to a rotavirus quantitative reverse-transcription PCR (reference assay). Rotavirus reference assay positive samples were P and G genotyped by sequencing the rotavirus VP4 and VP7 genes.

**Results:**

Overall agreement between the Luminex xTAG and the reference assay was excellent (kappa 0.93). The sensitivity and specificity was 88.2 % (95 % confidence interval [CI] 78.2–94.1) and 100 % (95 % CI 99.2–100), respectively. Of 76 rotavirus reference assay positive samples, 64 were successfully genotyped and the Luminex xTAG assay was able to detect all rotavirus genotypes present in the study.

**Conclusion:**

The Luminex xTAG assay proved a sensitive and highly specific tool to detect rotavirus and may aid clinicians and public health authorities in the diagnosis and surveillance of rotavirus.

## Background

Rotavirus gastroenteritis is a major public health threat responsible for a high disease burden in low-income countries [1]. Laboratory diagnosis of rotavirus gastroenteritis is traditionally accomplished using enzyme-immunoassays (EIA) and current EIA kits demonstrated a good overall performance [2]. However, PCR methods have proven their advantages over conventional methods mainly due to a higher analytical sensitivity [3, 4]. Commercial multiplex PCR panels became available recently allowing the simultaneous detection of several targets including rotavirus. These panels were already successfully evaluated in Europe, the USA, and more recently in Vietnam [5–7]. We applied the Luminex xTAG gastrointestinal pathogens panel (GPP) in a rural African setting and could demonstrate a high rate of positive stool samples [8]. The assay was applied to diagnose gastrointestinal pathogens in children with and without diarrhoea to evaluate the usefulness of the GPP. Overall the three most common pathogens were enterotoxigenic *Escherichia coli*, *Giardia lamblia*, and *Shigella* spp.. In particular, we could show an association with diarrhoea for rotavirus. However, there is only scant evidence on the diagnostic performance of commercial multiplex PCR panels using samples from tropical settings in general and to detect rotavirus in particular.

We aimed to evaluate the performance of the Luminex xTAG GPP multiplex PCR in comparison to a reference quantitative reverse-transcription PCR (qRT-PCR) to detect rotavirus in stool samples from Ghanaian children.

## Results

Among cases, 49/341 (14.4 %) tested positive using qRT-PCR, and 44/341 (12.9 %) tested positive using the GPP. In controls, 27/341 (7.9 %) were qRT-PCR positive, and 23/341 (6.7 %) were positive using the GPP assay. Comparable qRT-PCR Ct-values between cases (median Ct-value: 21.5, interquartile range [IQR]: 19.8; 25.1) and controls (median Ct-value: 22.0, IQR: 19.2; 27.0) were observed (*p* = 0.66). Rotavirus was detected more frequently in cases (*n* = 49, 14.4 %) compared to controls (*n* = 27, 7.9 %) using qRT-PCR (*p* = 0.010), and GPP (*n* = 44, 12.9 % versus *n* = 23, 6.7 %; *p* = 0.009), respectively.

Overall agreement between qRT-PCR and GPP was excellent (kappa 0,93). The test performance of the GPP in comparison to qRT-PCR is summarized in Table 1. In cases, the sensitivity of GPP was slightly higher (Table 1). The nine GPP false-negative results had a median qRT-PCR Ct-value of 30 (IQR: 28.8; 32.5) indicating low rotavirus loads. Of note, all nine samples tested rotavirus positive using the FTD viral gastroenteritis assay, suggesting that these samples were indeed GPP false-negatives. Five of these nine samples were from cases, and four from controls.

**Table 1:** Performance of the Luminex GPP in comparison to the rotavirus reference qRT-PCR assay among all study subjects (top panel) and cases only (bottom panel)

	TP, n	FP, n	FN, n	TN, n	Sensitivity % (95 % CI)	Specificity % (95 % CI)	kappa
All study subjects (*n* = 682)	67	0	9	606	88.2 (78.2–94.1)	100 (99.2–100)	0.93
Cases (*n* = 341)	44	0	5	292	89.7 (76.9–96.2)	100 (98.4–100)	0.94

*TP* true positive; *FP* false positive; *FN* false negative; *TN* true negative

**Fig. 1:**
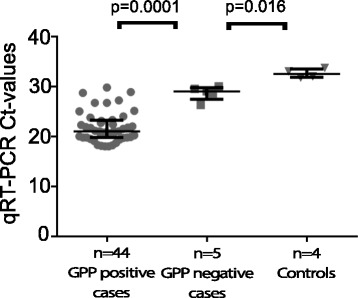
Distribution of qRT-PCR cyle threshold values. Shown are qRT-PCR cycle threshold values in GPP-positive and GPP-negative samples among cases and controls

There was a significant difference in qRT-PCR Ct-values between 44 GPP-positive (median Ct-value: 21, IQR: 19.8; 23.3) and five GPP-negative cases (median Ct-value: 29, IQR: 27.5; 29.8, *p* = 0.0001) (Fig. 1). Of the 76 qRT-PCR positive samples 64 (84 %) were rotavirus G and P genotyped. The GPP assay was able to detect all rotavirus genotypes present in the study (Table 2). Of note, we failed to genotype the nine GPP false-negative samples.

**Table 2:** Shown are results for the Luminex GPP assay according to rotavirus genotype and median Ct-value of qRT-PCR

Rotavirus genotype	No. of strains	Median qRT-PCR Ct-value, (IQR)	Luminex GPP positive, *n* = (%)
G1[P8]	25	20.3 (19.3–22.8)	25 (100)
G2[P4]	17	21 (19.3–22.6)	17 (100)
G2[P6]	12	20.4 (18.8–21.9)	12 (100)
G2[P-UD^a^]	7	25.1 (21.7–27.2)	7 (100)
G3[P6]	3	22.1 (20–23.1)	3 (100)
Total	64	21 (19.6–23.3)	64 (100)

*Abbreviations*: *IQR* interquartile range; *Ct* cycle threshold, *GPP* gastrointestinal pathogens

^a^ UD, undetermined

**Fig. 2:**
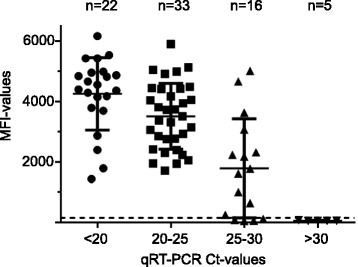
MFI-values according to qRT-PCR cycle threshold values. Shown are MFI-values using the GPP assay in four different groups with qRT-PCR Ct-values <20, 20–24, 25–29, and >30. The horizontal dotted line indicates the GPP threshold of 150 MFI for positivity

Comparable MFI-values using the GPP assay between cases (median MFI: 3875; IQR: 2791; 4795) and controls (median MFI: 3314, IQR: 2173; 4485) were observed (*p* = 0.33). Next, we analyzed GPP MFI-values in four different groups with qRT-PCR Ct-values <20 (median MFI: 4475, IQR: 3766; 4960), 20-25 (median MFI: 3505, IQR: 2583; 4414), 25–30 (median MFI: 1694, IQR: 145; 2882), and >30 (median MFI: 46, IQR: 44; 63), respectively (Fig. 2). There was a statistically significant difference between the groups with Ct-values ranging from 20–25, 25–30, and >30, respectively, using one-way ANOVA analysis. Only a weak negative correlation of −0.47 (95 % CI −6.64 to −0.26) was seen between qRT-PCR values and GPP MFI-values.

Finally, the number of co-detections with other pathogens as determined using the GPP did not differ significantly between cases (median 3, IQR: 2; 3) and controls (median 2, IQR: 1; 4), and between GPP-positive (median 2, IQR: 1; 3) and GPP-negative (median 2, IQR: 2; 4) samples. Overall the rate of co-detections was statistical significantly different between rotavirus GPP-positive [67/67 (100 %)] and GPP-negative [575/615 (93.5 %)] samples (Fishers exact test, *p* = 0.025). The distribution of pathogens among rotavirus GPP-positive and rotavirus GPP-negative samples is shown in Table 3. Of note, significantly more enterotoxigenic *Escherichia coli* (59 % versus 43 %, *p* = 0,0187), *Escherichia coli* O157 (15 % versus 3 %, *p* = 0,0029), and *Shigella* spp. (40 % versus 22 %, *p* = 0,0052) were detected among rotavirus GPP-negative samples compared to GPP-positive samples (Table 3).

**Table 3:** Number and percentage of co-detections among rotavirus GPP-positive and rotavirus GPP-negative samples

	Rotavirus GPP-positive, *n* = 67	Rotavirus GPP-negative, *n* = 615	
Pathogen	*n*=, (%)	*n*=, (%)	*p*-value*
Adenovirus 40/41	4 (6)	27 (4)	0,5338
Norovirus GI/GII	3 (4)	63 (10)	0,1884
*Campylobacter* spp.	19 (28)	212 (34)	0344
*Clostridium difficile toxin A/B*	1 (1)	3 (0,5)	0,3394
*Cryptosporidium* spp.	1 (1)	44 (7)	0,1137
*Entamoaba histolytica*	0 (0)	3 (0,5)	1
*Escherichia coli* LT/ST (ETEC)	29 (43)	362 (59)	*0,0187*
*Escherichia coli* O157	2 (3)	94 (15)	*0,0029*
*Escherichia coli* STEC (*stx*1/2)	1 (1)	20 (3)	0,7115
*Giardia lamblia*	28 (42)	325 (53)	0,0947
*Salmonella* spp.	6 (9)	84 (14)	0,3445
*Shigella* spp.	15 (22)	246 (40)	*0,0052*
*Yersinia enterocolitica*	0 (0)	0 (0)	1
*Vibrio cholerae*	0 (0)	0 (0)	1

*Fisher’s exact test

## Discussion

We could show an excellent agreement between the GPP and qRT-PCR to detect rotavirus in stool samples from Ghanaian children. The high specificity of the GPP is reassuring. The sensitivity of the GPP to detect rotavirus was slightly lower than reported previously [5, 9]. However, numbers of rotavirus positive samples were rather low in both studies [5, 9]. Another study from Vietnam used a comprehensive panel of samples and demonstrated a sensitivity of 92.2 % and specificity of 98.9 %, respectively, for rotavirus [7]. These data from an Asian tropical country are in line with our findings although different rotavirus qRT-PCR assays were used as a comparator. On the other hand, rare rotavirus genotypes or genetic variants which are prevalent in West Africa might undergo GPP detection, but warrants further studies [10]. As a limitation we could not sequence the qRT-PCR positive/GPP negative samples mainly due to low virus concentrations. Little left over material prevented further sequencing efforts.

Intriguingly, we detected less rotavirus shedding among controls using GPP and qRT-PCR compared to a recent study from Malawi [11]. Differences in herd immunity are possible reasons for this finding. However, rotavirus shedding using RT-PCR has been observed in different studies but the relevance remains unclear [12]. Subclinical infections or prolonged shedding after acute infections might play a role and deserve further studies. In light of the high sensitivity of the qRT-PCR and consistency with GPP results it is less likely that sensitivity issues played a role. Interestingly, the median qRT-PCR Ct-value of GPP-positive cases in our study is close to the Ct-value of 19.5 in diarrheal cases of another study supporting the notion that low Ct-values are associated with clinically relevant disease [11]. Of note, the results of the GPP are of qualitative nature only and quantitative results might provide a better resolution of PCR results and disease [13]. As a surrogate marker for rotavirus RNA concentrations we could show that MFI values of the GPP might provide useful semi-quantitative information. However, further studies are needed to establish reliable quantitative data using the GPP. Ultimately, studies are needed to appreciate the value of multiplex PCR in order to improve patient care. As a limitation of our study we did not address the diagnostic performance of all pathogens included in the GPP panel. Previous studies have shown a good overall performance but further studies using samples from Africa are needed.

## Conclusion

We evaluated the Luminex GPP assay to detect rotavirus using stool samples from Ghanaian children and could demonstrate that the Luminex GPP is a sensitive and highly specific tool for this purpose. The GPP is able to detect a broad range of rotavirus genotypes prevalent in Ghana and our results suggest that the assay can provide semi-quantitative data which requires further investigation. Thus, pending its implementation in resource-constrained African countries the GPP assay might provide a valuable tool in the detection and surveillance of rotavirus.

## Methods

Samples used in the current study were a subset of samples collected in the framework of a case-control study on causes of diarrhoea in children. The study was conducted at the children’s Outpatients Department (OPD) of the Agogo Presbyterian Hospital, a district hospital in the Ashanti region of Ghana [14]. For the current study, stool samples from children below 6 years of age visiting the hospital’s OPD between June 2007 and October 2008 were selected. Children with diarrhoea, defined as at least three loose stools within the last 24 h, served as cases. During the same period, children attending the OPD without gastrointestinal symptoms were recruited as controls. A total of 682 samples were selected for this study. Of these, 341 samples were from cases and another 341 were from controls.

Immediately after stool collection, the samples were frozen at −20 °C and shipped on dry ice to Germany for further analyses as described elsewhere [14]. Following nucleic acid extraction from 200 mg stool samples were analyzed using the GPP (Luminex Corporation, Austin, TX) according to the manufacturer’s instructions. The results for rotavirus are reported in median fluorescence intensity (MFI) values from each sample. A published rotavirus qRT-PCR was used as the reference assay [15]. Briefly, a qRT-PCR targeting the NSP3 gene was used to detect rotavirus nucleic acids. A 25-μl reaction contained 5 μl of prepared nucleic acids (the same as used for the GPP), 2× AgPath-ID One-Step RT-PCR buffer (Thermo Fisher, Darmstadt, Germany), 0.4 μM primer JVKF (CAGTGGTTGATGCTCAAGATGGA; TIB-Molbiol, Berlin, Germany), 0.4 μM primer JVKR (TCATTGTAATCATATTGAATACCCA, TIB-Molbiol), 0.2 μM probe JVKP (ACAACTGCAGCTTCAAAAGAAGWGT). Probe JVKP was labeled with 5′ FAM and a 3′ nonfluorescent quencher (TIB-Molbiol). The cycling conditions in an ABI Prism 7500 machine (Thermo Fisher) were as follows: 50 °C for 15 min, 95 °C for 10 min, and 45 cycles of 95 °C for 10 s and 60 °C for 35 s. The data were analyzed with the Sequence detector software V 2.0.6 (Thermo Fisher). Positive and no-template controls were included in each PCR run.

The analytical sensitivity of the qRT-PCR was 12.5 in vitro-transcribed RNA copies per reaction [95 % confidence interval (CI) 9.6 to 20.9 RNA copies per reaction] as determined using probit analysis. The rotavirus strains were G and P genotyped by sequencing according to Iturizza-Gomara et al. [16]. The Fast Track Diagnostics (FTD) viral gastroenteritis kit (Junglingster, Luxemburg) was used to analyze discrepant results between GPP and qRT-PCR. A sample was considered GPP false-positive if both the qRT-PCR and the viral gastroenteritis kit yielded a negative rotavirus result.

All statistical analyses were done using the GraphPad Prism software package (GraphPad, San Diego, CA, USA). Median cycle threshold (Ct)-values and MFI-values between two groups was compared using Wilcoxon rank sum test. Differences in the detection frequency of rotavirus using GPP and qRT-PCR in cases and controls were assessed with Fisher’s exact test. A *p*-value of <0.05 was considered statistically significant. Correlation between qRT-PCR Ct-values and GPP MFI-values was assessed using the Pearson correlation coefficient. The Committee on Human Research, Publications and Ethics, School of Medical Science, Kwame Nkrumah University of Science and Technology, Kumasi, Ghana, approved the study design and the informed consent procedure.

## Abbreviations

CI, confidence interval; Ct-value, cycle threshold value; EIA, enzyme-linked immunosorbent assay; GPP, gastrointestinal pathogens panel; IQR, interquartile range; MFI, mean fluorescence intensity; OPD, Outpatients Department; qRT-PCR, quantitative reverse-transcription polymerase chain reaction.
